# Using Generative AI in Learning and Students’ Innovative Behavior: A Dual-Path Examination Based on the UTAUT Model

**DOI:** 10.3390/bs16061002

**Published:** 2026-06-16

**Authors:** Lingyi Huang, Wenhao Luo

**Affiliations:** School of Economics and Management, North China University of Technology, No. 5 Jinyuanzhuang Road, Shijingshan District, Beijing 100144, China; 23102010214@mail.ncut.edu.cn

**Keywords:** use of GAI, innovative behavior, effort expectancy, performance expectancy, growth mindset

## Abstract

The rapid development of generative artificial intelligence (GAI) has exerted extensive and far-reaching impacts on college students’ learning, making it a topic worthy of in-depth investigation. This study aims to explore the impact of GAI usage on college students’ innovative learning behaviors, drawing on the theoretical framework of the Unified Theory of Acceptance and Use of Technology (UTAUT). Specifically, the research explores the mediating mechanisms of effort expectancy and performance expectancy, as well as the moderating role of growth mindset in this process. Based on a sample of 430 Chinese college students recruited from diverse academic majors, the proposed moderated mediation model is empirically examined through latent structural equation modeling analysis. The results indicate that using GAI in learning significantly enhances students’ perceptions of effort expectancy and performance expectancy, thereby fostering their subsequent innovative behavior. Notably, the findings reveal that while performance expectancy mediates the relationship between GAI usage and innovative behavior, a growth mindset weakens this indirect pathway. The practical implications of this study are also discussed for both students and universities.

## 1. Introduction

At present, GAI is extensively applied in educational scenarios, exerting a profound impact on college students’ learning (Chan & Hu, 2023). According to the AI in Education Statistics 2026 (Global Trends & Facts) report by Demand Sage, approximately 54% of students use AI on a daily or weekly basis, while 86% of students use multiple AI tools worldwide. By the end of 2025, the market value of AI education has increased to $7.57 billion compared to last year’s $5.47 billion. Nevertheless, the effectiveness of using GAI in learning has sparked various opinions (Ivanov et al., 2024). Proponents of GAI argue that the use of GAI could foster creativity and innovation for students (Law, 2024; Zhou & Peng, 2025; Ma et al., 2024), while critics hold the view that it may lead to students’ over-reliance on GAI, thereby reducing their opportunities for independent thinking and exploration and ultimately impeding the development of their creative thinking (Liu et al., 2024; Zhai et al., 2024). It has been widely reported on social media platforms such as YouTube that some professors yell at their students for using GAI without any thought. In work settings, researchers have also conducted studies on the impact of GAI on employees (S. Sun et al., 2025; Jia et al., 2024; Yin et al., 2024), suggesting that GAI exerts a double-edged-sword effect on employees’ innovative behavior. Despite the potential implications, such studies are rooted in organizational contexts that are fundamentally different from those on college students.

In previous studies, several factors have been shown to influence innovative behaviors, such as the satisfaction of psychological needs and self-esteem threats (Ji et al., 2025; L. Luo et al., 2025). In the context of higher education, studies have explored technological tools such as GAI that improve learning outcomes, increase learning opportunities, and offer personalized learning experiences (Shahzad et al., 2025). Recently, Ji et al. (2025) found that the emergence of GAI has significantly transformed learning patterns and innovative approaches, yet the underlying mechanism linking its adoption to innovative behaviors remains unknown. In this vein, the study aims to examine the relationship between the use of GAI and innovative behaviors, as well as exploring the mediating and moderating mechanisms.

To address the research questions, the Unified Theory of Acceptance and Use of Technology (UTAUT, Venkatesh et al., 2003; Y. Luo & Day, 2026) is introduced to investigate the mechanism through which GAI use in learning influences college students’ innovative behavior. The UTAUT identifies four core determinant variables, including performance expectancy, effort expectancy, social influence, and facilitating conditions, which together account for individuals’ behavioral intention and technology use behavior (Venkatesh et al., 2003). Most existing studies apply UTAUT to explore how perceptual factors drive use behavior (Xu et al., 2025; Amin et al., 2025). Rather than merely focusing on individuals’ adoption decisions, this study broadens the application of UTAUT and centers on the in-depth impacts of long-term technology usage on students’ personal development. With the continuous usage of GAI, students will change their usage cost and comprehensive judgments of tool value, and further shape students’ cognitive mindset and behavioral patterns, which may include the development of innovative behaviors. Therefore, the study takes effort expectancy and performance expectancy as mediating variables to clarify the internal relationship between the use of GAI and students’ innovative behaviors.

Furthermore, it is unlikely that all college students will similarly reap the same benefits of using GAI in learning. According to UTAUT (Venkatesh et al., 2003), individuals’ perceptions of effort expectancy and performance expectancy are moderated by factors like age, gender and experience, leading students to differ in their responses to GAI. For instance, some students may resist relying on GAI, as it is likely that they will develop habits related to procrastination and memory loss, leading to dampened academic performance (Abbas et al., 2024). Therefore, how students respond to GAI plays a necessary role in understanding its impacts on innovative behaviors and should be taken into account. To explain such individual differences, the growth mindset is posited to act as a key boundary of the aforementioned mechanism. That is to say, when using GAI in learning, students with a growth mindset are more likely to perceive effort expectancy and performance expectancy, and subsequently engage in more innovative behaviors.

In conclusion, this research aims to make three contributions to the literature. First, the scope of GAI usage research is expanded by focusing on its influence on students’ innovative behavior, which represents sustained behavioral outcomes in learning contexts. Second, two key expectancies are introduced as cognitive mechanisms, revealing how they mediate the link between the use of GAI and innovative behavior. Third, a growth mindset is identified as an important boundary of expectancies, clarifying when expectancies are more likely to promote innovative behavior among students.

## 2. Theory and Hypothesis Development

### 2.1. Unified Theory of Acceptance and Use of Technology

UTAUT is an integrated theoretical framework synthesized from classic models, including the Theory of Reasoned Action (TRA), the Technology Acceptance Model (TAM), and other mainstream technology adoption theories (Venkatesh et al., 2003). Specifically, performance expectancy, effort expectancy, and social influence jointly shape users’ behavioral intention, which in turn drives their use behavior. By contrast, facilitating conditions exert a direct effect on usage behavior. In addition, personal attributes, including gender, age, experience and voluntariness of use, moderate the above relationships and generate distinct technology adoption across different individuals and situations. Accordingly, UTAUT offers a well-established analytical framework for understanding the drivers of technology adoption.

UTAUT thus provides a solid theoretical foundation for exploring the intrinsic relationship between students’ use of GAI and their innovative behaviors. The UTAUT primarily explains the influencing factors of individuals’ technology adoption decisions, in which effort expectancy and performance expectancy serve as the antecedents of use behavior (Venkatesh et al., 2003). However, this logic primarily reflects the adoption stage and focuses on how expectancy beliefs influence subsequent technology use. Following actual technology use, expectancy beliefs may no longer remain fixed. Research on information systems continuance indicates that users continually reassess and update their beliefs through actual interactions with a technology (Bhattacherjee, 2001; Hong et al., 2006). Specifically, Bhattacherjee (2001) argued that post-adoption perceptions and continuance-related evaluations may be shaped through confirmation processes emerging from actual use. In other words, experiences gained through technology use may provide new information that reinforces, refines, or revises users’ prior expectations. To further explain how expectancy beliefs evolve after actual use, Venkatesh et al. (2011) argued that pre-usage beliefs serve as anchors for post-usage beliefs and that effort expectancy becomes well-formed only after hands-on experience. This suggests that actual usage experiences help reduce uncertainty surrounding users’ initial beliefs and contribute to the further development of expectancy perceptions (Venkatesh, 1999). Taken together, these studies suggest that expectancy beliefs are not merely antecedents of technology use but may also evolve through sustained interaction with the technology in question. This view complements the original UTAUT perspective by suggesting that, although effort expectancy and performance expectancy influence technology use during the adoption stage, actual usage experiences may also contribute to the formation and refinement of these beliefs in post-adoption contexts. Therefore, students’ use of GAI may shape the development of effort expectancy and performance expectancy over time.

Meanwhile, the UTAUT highlights that individual traits can exert different effects on the overall paths (Venkatesh et al., 2003). This core viewpoint also offers theoretical support for introducing the growth mindset as the moderating variable in this study. Students with different levels of growth mindset demonstrate distinct sensitivity in cognitive transformation and behavioral output, which directly alters the impact strength of effort expectancy and performance expectancy on innovative behaviors.

**Figure 1:**
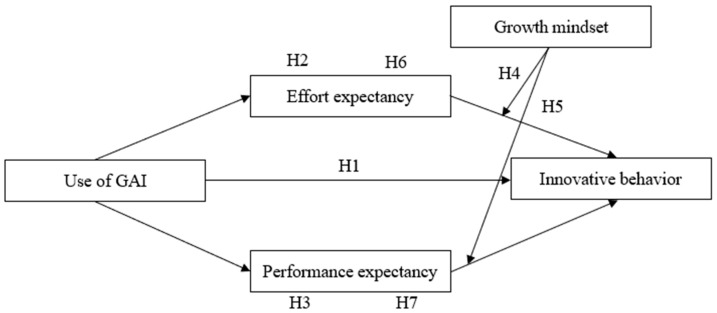
The theoretical model.

In summary, grounded in the UTAUT, the study establishes a pathway from the use of GAI to college students’ innovative behaviors through performance expectancy and effort expectancy, while incorporating growth mindset as a moderating factor (see Figure 1). Consistent with the assumptions of UTAUT, this study highlights that technology-related cognition remains an important mechanism underlying individuals’ interactions with technology, while suggesting that such cognition may continue to develop throughout the technology use process.

### 2.2. Use of GAI and Innovative Behavior

Creativity is defined as the ability to generate novel and useful ideas (Sternberg, 1999). UNESCO has highlighted that cultivating creative citizens will become a core primary goal of education in the AI era (Miao & Shiohira, 2024). Innovative behavior refers to the creativity and uniqueness demonstrated by students in the learning process, including exploring and sharing new ideas and solutions, proposing new perspectives or methods, and critically analyzing and improving existing knowledge (Barak et al., 2020). It is essential for students’ personal growth, which can boost learning and work efficiency and facilitate long-term development and career progress (Genco et al., 2012; Keinänen et al., 2018). Since the cultivation of innovative behavior necessitates a supportive environment, the advent of GAI provides favorable conditions for students to develop innovative behavior (Filippi, 2023). Therefore, it is better to explore the underlying relationship between the use of GAI and innovative behavior and formulate corresponding research hypotheses.

As students use GAI, they move beyond basic knowledge acquisition to explore ideas, elaborate on issues and refine solutions. Rather than a passive tool, GAI can act as an interactive partner for learners (Chiou & Lee, 2023). Such interaction encourages in-depth reflection, questioning and exploration, which strengthens students’ critical thinking (Suriano et al., 2025). Gonsalves (2026) also found that this evaluative thinking drives students to deconstruct and reorganize GAI outputs. Through iterative revision and optimization of these contents, students develop original viewpoints, while sustained creative practice further cultivates innovative thinking and behaviors.

In addition, GAI can also tailor learning content and pathways to individual needs, allowing students to regulate their own progress and exercise greater control over learning activities, which may strengthen their perceived autonomy (Ryan & Deci, 2008; Reeve, 2006). Perceived autonomy refers to the extent to which students have discretion in accessing learning resources, structuring their learning process, and managing their learning time (Gagné & Deci, 2005; Xia et al., 2022). Students with higher perceived autonomy may be more likely to actively seek new knowledge for personal growth and engage in creative problem-solving (L. Luo et al., 2025). Recent studies have shown that autonomous learning environments encourage students to experiment with diverse approaches and generate alternative solutions during task completion, which could contribute to the development of innovative behavior (Tsai, 2013). Drawing on the above evidence, this study aims to explore how GAI use relates to students’ innovative behavior.

**H1.**  *Use of GAI is positively associated with innovative behavior.*

### 2.3. The Mediating Role of Effort Expectancy

Effort expectancy refers to the perceived ease of using a technology (Venkatesh et al., 2003). It encompasses factors such as system complexity and perceived ease of use, which influence user acceptance (Strzelecki et al., 2024). Within the UTAUT framework, effort expectancy is traditionally conceptualized as a determinant of technology adoption (Venkatesh et al., 2003). Prior research suggests that effort expectancy beliefs may be subject to change after actual system use, as such beliefs become well-formed through hands-on experience (Venkatesh & Davis, 1996; Venkatesh et al., 2011). Accordingly, examining effort expectancy as a consequence of students’ GAI use is theoretically justified. In this study, effort expectancy serves as a critical cognitive mediator through which students’ use of GAI may influence their innovative behaviors.

College students engaging with GAI in learning can access personalized learning materials, tailored learning tasks, and adaptive learning support (Chan & Hu, 2023). For students, the accessible and low-threshold functional support may be closely associated with positive effort expectancy toward GAI (Y. Sun et al., 2026b). These resources are readily available without complicated operations, which supports the formation of students’ effort expectancy. As stated by Mwakapesa (2025), this positive effort expectancy further reduces students’ cognitive load during GAI interaction and eases mental burden in learning. Driven by such low usage barriers, students tend to assign repetitive routine tasks to GAI rather than spending considerable time on inefficient and tedious processes, thereby potentially reducing their perceived time pressure when completing coursework. Perceived time pressure refers to the perception that individuals lack sufficient time to complete tasks (Maruping et al., 2015) and it also reflects a state of external dysregulated context (De la Fuente et al., 2022), a situation that impairs students’ academic control (Y. Li & Jiang, 2025). De la Fuente et al. (2022) demonstrated that dysregulated learning contexts are typically associated with elevated stress levels and diminished learning adaptability. Therefore, as perceived time pressure eases, students’ external learning environment shifts from imbalance to a more supportive condition (De la Fuente et al., 2022) and elicits positive emotions (Wu & Yu, 2022). When in a positive emotional state, students experience less stress and fewer distractions, exhibit more flexible and creative thinking patterns, and can allocate their cognitive resources more intensively and effectively to creative thinking and innovative behaviors (Xie et al., 2022; L. Yang & Zhao, 2024; Ma et al., 2024).

Furthermore, GAI exhibits distinct anthropomorphic interactive features. Anthropomorphism refers to the attribution of human traits to nonhuman entities (Epley et al., 2007). Previous research has shown that when the entity is anthropomorphized, the psychological distance between users and that entity decreases (X. Li & Sung, 2021). Building on this perspective, Uysal et al. (2022) suggested that reduced psychological distance can lead users to perceive GAI as more socially approachable rather than merely a cold and complex functional tool. This approachable interaction may, in turn, diminish perceived operational difficulty and lower cognitive load, making the system feel more effortless to use (Blut et al., 2021). Under such conditions, students could develop stronger effort expectancy toward GAI. High effort expectancy can further reduce hesitation toward trial and exploration, encouraging more exploratory engagement with GAI (Duong et al., 2024). The openness to exploration exposes students to diverse perspectives and alternative ways of thinking, thereby contributing to cognitive flexibility and supporting innovative behaviors (X. Chen & Xiao, 2025).

**H2.**  *Effort expectancy mediates the relationship between the use of GAI and innovative behavior.*

### 2.4. The Mediating Role of Performance Expectancy

Performance expectancy refers to the perceived usefulness or the degree to which individuals believe that utilizing a system will enhance their job or learning performance (Venkatesh et al., 2003). Beyond its established role in technology adoption (Yakubu et al., 2025; Venkatesh et al., 2003), perceptions of usefulness may change following actual system use, as users gain an updated understanding of the performance value of the technology (Bhattacherjee, 2001). This suggests that students’ performance expectancy may continue to evolve as they accumulate experience with GAI. Therefore, performance expectancy is considered a cognitive mechanism linking students’ GAI use to their innovative behaviors.

When college students use GAI, they can clearly perceive a high level of informativeness, as the tool provides substantial support for their academic tasks (Kirmani, 2022). The practical assistance provided by GAI enables students to directly experience improvements in learning efficiency and task performance, thereby gradually forming a stronger performance expectancy toward GAI. For example, Noy and Zhang (2023) found that the use of GAI significantly improved individuals’ productivity and the quality of knowledge-intensive work outputs, implying that students are more likely to perceive the performance-oriented value of GAI during task completion. Beyond productivity gains, the ability of GAI to deliver high efficiency and demonstrate unexpected outputs may evoke awe among users (L. Zhao et al., 2025). Fredrickson (2001) suggested that positive awe can broaden individuals’ thought–action repertoires and encourage exploratory thoughts and behaviors. Prior research has also noted that intrinsically motivated individuals are more likely to engage in experimentation, divergent thinking, and exploratory problem solving (Amabile et al., 1996). Building on these links, as interactions with GAI become more engaging and enjoyable, students may gradually develop stronger intrinsic motivation toward AI-assisted learning and become more willing to break through fixed thinking patterns and explore novel approaches, ultimately promoting innovative behaviors.

In addition, college students gain creative inspiration, which not only enhances the novelty and practicality of their outputs (Doshi & Hauser, 2024), but also assists them in generating, organizing, and refining ideas more effectively for academic tasks (Noy & Zhang, 2023). According to existing research, AI-assisted systems can support users in processing information from multiple perspectives and exploring alternative solutions during complex tasks, thereby facilitating their creative exploration and idea development (Kasneci et al., 2023). Through the experiences, students gradually recognize the practical value of GAI and develop a high level of performance expectancy. Driven by this awareness of GAI’s value, students are more inclined to maintain active engagement and intentional control during use, rather than passively relying on AI-generated outputs. Guo et al. (2025) similarly observed that individuals who perceive GAI as valuable and supportive tend to demonstrate stronger initiative and proactive participation during AI-assisted tasks. Faraj et al. (2018) also emphasized that while GAI can enhance decision-making efficiency and provide relevant support, achieving effective outcomes still relies on individuals’ sustained engagement and independent judgment throughout the human–AI interaction process. Such intentional engagement enables students to adjust AI-generated content based on their own understanding, which reduces the anchoring effect caused by passively accepting GAI perspectives. As a result, students may engage in deeper creative thinking and further cultivate innovative behavior.

**H3.**  *Performance expectancy mediates the relationship between the use of GAI and innovative behavior.*

### 2.5. The Moderating Role of Growth Mindset

Growth mindset is defined as a belief that abilities are malleable and can be developed through effort (Dweck, 2006). Building on the UTAUT framework, which has established performance expectancy and effort expectancy as key constructs in technology use contexts, we extend this perspective by examining how these expectancy factors relate to subsequent innovative behavior, and view growth mindset as a boundary condition shaping the relationships.

First, growth mindset moderates the relationship between effort expectancy and innovative behavior. When college students possess a high level of growth mindset, they believe that technological ability can be gradually developed through continuous learning and effort (Pybus & Gillan, 2015). Therefore, even when using unfamiliar GAI functions, they are more likely to view operational challenges as opportunities for learning and skill development rather than as threats to their competence. Research has shown that individuals with a stronger growth mindset are more likely to adopt mastery-oriented goals, actively embrace challenges, and persist in exploratory learning activities (Burnette et al., 2013; Yeager & Dweck, 2020). Accordingly, when students perceive GAI as easy to use, they are more likely to transform the low operational barriers into exploratory engagement with GAI. Through ongoing exploration, students can facilitate idea generation and develop more flexible approaches to problem solving, which are important antecedents of innovative behavior (Hirst et al., 2009; Tierney & Farmer, 2011). In contrast, students with a lower level of growth mindset tend to fear failure, persist less in learning new skills, and exhibit poor self-regulation when solving problems (Dweck, 1999; Stipek & Gralinski, 1996). This avoidance-oriented regulation reduces participation in challenging, uncertain learning activities and iterative trial-and-error exploration (Sisk et al., 2018; Yeager et al., 2019). When using GAI, such individuals rarely explore advanced or unfamiliar functions, even if they find the tool user-friendly. From this perspective, perceived ease of use does not automatically translate into willingness to engage in exploratory interaction. Accordingly, effort expectancy is less likely to lead to innovative behavior under conditions of a lower growth mindset. Overall, the positive effect of effort expectancy on innovative behavior is stronger under conditions of a high-growth mindset.

Second, growth mindset also moderates the relationship between performance expectancy and innovative behavior. Students with a high-growth mindset focus more on long-term self-improvement and their own abilities, and tend to adopt strategies that foster personal progress (Blackwell et al., 2007). For example, they evaluate learning outcomes based on capability growth rather than short-term performance gains. They further view challenges as opportunities for learning and growth, and maintain greater persistence in demanding tasks (Chow & To, 2025). In particular, such persistence stems from a learning orientation, which prioritizes learning and competence improvement over immediate task outcomes (Senko et al., 2011). Consequently, they place little emphasis on the immediate improvements brought by GAI. These superficial gains fade once GAI support is removed and fail to reflect genuine growth in core abilities such as creativity (Darvishi et al., 2024; Guo et al., 2025), and thus can hardly translate into sustained innovative behavior. Conversely, students with a low-growth mindset perceive achievement contexts as evaluative situations and tend to be oriented toward performance goals (Dweck & Leggett, 1988; Elliot & McGregor, 2001). For example, Mueller and Dweck (1998) found that students holding performance goal orientations tend to focus on external markers of success and failure, and judge their performance by tangible outcomes instead of learning progress. Such an outcome-focused orientation is known to diminish engagement in deep learning processes, with greater weight placed on task completion and efficiency rather than knowledge development (Elliot & Church, 1997). As a result, performance expectancy tends to boost task engagement and involvement when students use GAI that facilitates visible performance gains efficiently. Therefore, the effect of performance expectancy on innovative behavior is more significant for students with a low-growth mindset.

**H4.**  *Growth mindset strengthens the relationship between effort expectancy and innovative behavior, so the relationship is stronger for college students with a higher level of growth mindset.*

**H5.**  *Growth mindset weakens the relationship between performance expectancy and innovative behavior, so the relationship is stronger for college students with a lower level of growth mindset.*

Taken as a whole, students who use GAI in learning are more likely to form effort expectancy and performance expectancy. Compared with students with a lower level of growth mindset, those with a higher level of growth mindset are more likely to strengthen the effect of effort expectancy on innovative behavior, while weakening the effect of performance expectancy on innovative behavior.

**H6.**  *Growth mindset strengthens the relationship between the use of GAI and innovative behavior via effort expectancy, so the indirect effect is stronger for college students with a higher level of growth mindset.*

**H7.**  *Growth mindset weakens the relationship between the use of GAI and innovative behavior via performance expectancy, so the indirect effect is stronger for college students with a lower level of growth mindset.*

## 3. Method

### 3.1. Sample and Procedures

For the purposes of the study, we distributed questionnaires to college students across diverse majors. Participant recruitment relied on Credamo, a professional online platform widely used for academic research in China (e.g., Z. Yang & Tian, 2026; Z. Chen et al., 2024; Y. Sun et al., 2026a). All respondents received clear information about study procedures, along with explicit guarantees of data confidentiality and anonymity. Those who completed the questionnaire were rewarded with RMB 5 yuan (approximately US$1). Given the average total time required to complete the questionnaire (8.43 min), this incentive was appropriate for college students. The questionnaire primarily examined college students’ GAI usage behaviors, relevant experiences and impacts of GAI, as well as their learning status, innovative behavior, and demographic information.

We received 500 responses, of which 430 were complete and valid. Responses were excluded if participants failed attention check items, left sections unfinished, or required an unreasonably long or short amount of time to complete the questionnaire. To assess non-response bias, this study ran independent sample t-tests between the final sample and the excluded sample, as well as compared the early 50% and late 50% of respondents (Armstrong & Overton, 1977). Test outcomes revealed no significant demographic differences between the grouped samples, which confirmed that non-response bias exerted little influence on the current research.

The valid sample presents the following demographic characteristics. Specifically, 50.23% of respondents were female, and 61.16% of participants belonged to the age group of 18 to 22 years, while 98.84% of the sample held at least a bachelor’s degree. Participants came from various academic disciplines, including engineering (30.7%), management (16.98%), economics (11.4%), natural science (13.49%), education (9.3%), literature (6.98%), medicine (5.81%), law (3.26%), philosophy (0.7%), agriculture (1.16%) and history (0.23%). Participants reported diverse GAI tool usage, with adoption rates covering DeepSeek (89.3%), Kimi (55.58%), Doubao (45.81%), ChatGPT (41.4%), ERNIE Bot (29.77%) and other tools (2.79%).

### 3.2. Measures

All of the measures in this study were adapted from previous research, with full items provided in Appendix A. Given that the original scales were developed in English, Brislin’s (1986) translation and back-translation procedures were used to create Chinese versions. Every item was assessed using a 7-point Likert scale, with scores ranging from 1 (strongly disagree) to 7 (strongly agree).

*Use of GAI*. It was measured with an 11-item scale developed by Y. Li et al. (2024) and Abbas et al. (2024). The scale developed by Y. Li et al. (2024) was tailored to the specific learning context of Chinese college students, capturing the unique situational and behavioral characteristics of their GAI use. Meanwhile, the scale proposed by Abbas et al. (2024) has demonstrated sound reliability and validity. By combining these two scales, the measure aligns with the experience of local students and provides a comprehensive assessment of students’ GAI application behaviors. A sample item was “I use generative AI for my academic activities” and the Cronbach’s α was 0.746.

*Innovative behavior.* It was measured with a 6-item scale developed by Scott and Bruce (1994). This scale covers the complete process of innovative behavior including idea generation, promotion and implementation. Given the shared characteristics of such behaviors across groups, the scale was adapted for student samples. A sample item was “Develops adequate plans and schedules for the implementation of new ideas” and the Cronbach’s α was 0.863.

*Growth mindset.* It was measured with an 8-item scale developed by Sigmundsson and Haga (2024) and Xiao et al. (2026). Both instruments have shown sound applicability across broad age groups, demonstrating that growth mindset operates through consistent underlying mechanisms across different populations. Items from the two scales were selected and revised to suit the context of college students in the study. A sample item was “Trying new things is a way to grow your intelligence” and the Cronbach’s α was 0.891.

*Effort expectancy.* It was measured with a 4-item scale developed by Cao et al. (2021). A sample item was “Learning how to use generative AI is easy for me” and the Cronbach’s α was 0.637.

*Performance expectancy.* It was measured with a 5-item scale developed by Cao et al. (2021). A sample item was “I find generative AI useful in my decision-making,” and the Cronbach’s α was 0.665.

*Control variables.* Following prior studies (Sokić et al., 2021; Tsai, 2013; Cheung et al., 2003), gender, age, educational level and major were chosen as control variables.

### 3.3. Analytical Strategy

First, a series of confirmatory factor analyses (CFAs) was conducted in Mplus 8.3 (Muthén & Muthén, 2017) to assess the fit of the theoretical model, thereby evaluating measurement validity and detecting common method bias. Second, the research used SPSS 27.0 to calculate descriptive statistics, bivariate correlations and Cronbach’s alphas of the study variables. Third, latent structural equation modeling was performed in Mplus 8.3 (Muthén & Muthén, 2017) to test all the hypotheses.

## 4. Results

### 4.1. Common Method Bias and the Discriminant Validity

Given that all data were self-reported, potential common method bias might be present. The unmeasured latent method construct approach was employed to address this issue, using a six-factor model that included a common method variance (CMV) factor to examine the impact of CMV (Podsakoff et al., 2003). As shown in Table 1, the six-factor model demonstrated better fitting indices than the theoretical model (the five-factor model). A variance decomposition analysis was also performed to partition variance into trait, method, and random error components (Williams et al., 1989). Results showed that the five traits (i.e., the five variables in this model) contributed 36.16% to total variance, the CMV factor accounted for only 9.18%, and random error accounted for 54.66%. The percentage of variance explained by the method factor was much lower than both the variance explained by the trait factors and the average method variance (27%) proposed by Williams et al. (1989). Following the recommendations from prior studies (Choi & Chen, 2007; Diefendorff & Mehta, 2007), these results indicate that CMV was not a major concern in the data analysis.

A set of CFAs was conducted to examine the construct validity. Considering the item–sample ratio, the well-established item parceling approach was adopted to model estimation (Landis et al., 2000). Three-item parcels were formed for the use of GAI, and two-item parcels for growth mindset. As depicted in Table 1, the five-factor structure demonstrated a better fit (*χ*^2^ = 347.771; *df* = 160; root mean square error approximation (RMSEA) = 0.052; standardized root mean square residual (SRMR) = 0.052; comparative fit index (CFI) = 0.927; Tucker–Lewis index (TLI) = 0.913) than other alternative models. Factor loadings ranged from 0.424 to 0.887, suggesting that the five variables can be empirically discriminated from each other.

### 4.2. Descriptive Statistics

Table 2 presents the descriptive statistics and correlations of all variables. As shown in Table 2, all core variables were significantly correlated with each other. Results showed that the use of GAI was correlated to effort expectancy (r = 0.286, *p* < 0.01), performance expectancy (r = 0.391, *p* < 0.01) and innovative behavior (r = 0.208, *p* < 0.01); innovative behavior was correlated to effort expectancy (r = 0.313, *p* < 0.01) and performance expectancy (r = 0.322, *p* < 0.01); growth mindset was correlated to effort expectancy (r = 0.176, *p* < 0.01), performance expectancy (r = 0.181, *p* < 0.01) and innovative behavior (r = 0.443, *p* < 0.01). These results provided some preliminary support for the following hypothesis testing.

### 4.3. Hypothesis Testing

The hypotheses were tested using latent structural equation modeling in Mplus 8.3 (Muthén & Muthén, 2017), and the results are presented in Table 3 and Table 4. Hypothesis 1 proposed that the use of GAI can facilitate students’ innovative behavior. As shown in Table 3, the relationship between the use of GAI and innovative behavior was not statistically significant (*b* = −0.19, *p* > 0.05), thus not supporting Hypothesis 1. Although the direct effect was non-significant, the finding did not necessarily invalidate the proposed framework. Instead, it suggested that the influence of GAI use on innovative behavior may be more complex and operate indirectly through students’ cognitive evaluations of AI technologies. In educational contexts, simply using GAI tools may not automatically translate into innovative actions unless students perceive such technologies as useful and manageable in supporting academic tasks and problem-solving activities. This result further highlights the importance of examining the mediating mechanisms underlying the relationship between the use of GAI and innovative behavior.

To test the mediating roles of effort expectancy (Hypothesis 2) and performance expectancy (Hypothesis 3), the study used Mplus 8.3 (Muthén & Muthén, 2017) to calculate the 95% confidence intervals (CIs) of indirect effects following the method proposed by Preacher and Selig (2012). The results indicated that 0 was excluded from the 95% confidence intervals, demonstrating significant mediation effects. Specifically, the indirect effect of using GAI on innovative behavior through effort expectancy was significant (indirect effect = 0.157, standard error (SE) = 0.053, 95% confidence interval (CI) = [0.053, 0.261]). This finding indicated that students who perceive GAI as easier to use were more likely to engage in innovative activities, as lower perceived complexity might increase confidence in applying GAI-supported learning approaches. Similarly, the indirect effect of using GAI on innovative behavior through performance expectancy was also significant (indirect effect = 0.288, standard error (SE) = 0.078, 95% confidence interval (CI) = [0.135, 0.440]). The relatively stronger indirect effect through performance expectancy suggested that students’ perceptions regarding the usefulness and effectiveness of GAI played an important role in promoting their innovative behavior. When they believe that GAI could improve academic performance and learning efficiency, they might become more willing to experiment with new ideas and adopt innovative problem-solving strategies. Therefore, Hypothesis 2 and Hypothesis 3 were supported.

To test the moderating role of growth mindset on the relationships between effort expectancy (Hypothesis 4) and performance expectancy (Hypothesis 5) and innovative behavior, interaction terms using the latent value of effort expectancy, performance expectancy and growth mindset were included in the model. The Bayes estimation results demonstrated that the interaction between effort expectancy and growth mindset did not significantly predict innovative behavior (*b* = 0.133, *p* > 0.05), indicating that the relationship between effort expectancy and innovative behavior remained relatively stable across different levels of growth mindset. This finding suggests that perceiving GAI as easy to use might generally encourage innovative behavior among students regardless of their level of growth mindset. In contrast, the interaction between performance expectancy and growth mindset significantly predicted innovative behavior (*b* = −0.872, *p* < 0.01).

**Figure 2:**
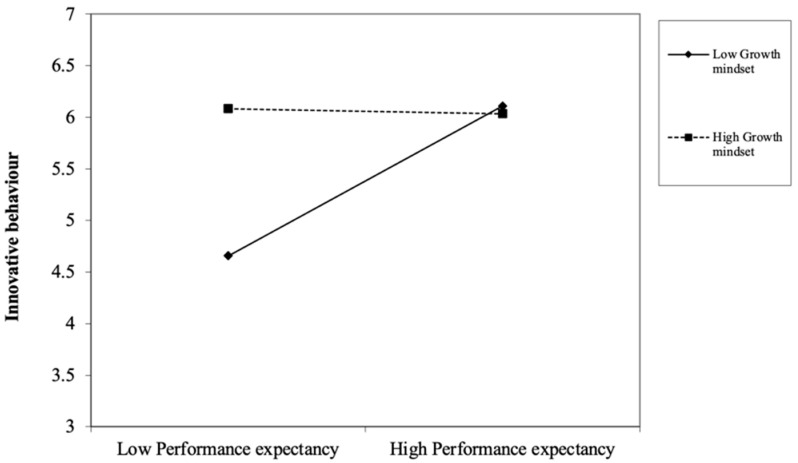
The interaction between performance expectancy and growth mindset on innovative behavior.

Following the approach proposed by Aiken and West (1991), the interaction effects are plotted in Figure 2. The simple slope analyses showed that for college students with a higher level of growth mindset, the effect of performance expectancy on innovative behavior was weaker and not statistically significant (simple slope = −0.047, *p* > 0.05). Conversely, for college students with a lower level of growth mindset, the effect of performance expectancy on innovative behavior was significantly stronger (simple slope = 1.440, *p* < 0.01). As illustrated in Figure 2, innovative behavior among students with a higher level of growth mindset remained stable across different levels of performance expectancy, whereas students with a lower level of growth mindset showed a more marked increase in innovative behavior as performance expectancy rose. This finding suggested that students with lower levels of growth mindset might be more influenced by performance-related outcomes associated with the use of GAI. Therefore, Hypothesis 4 was not supported and Hypothesis 5 was supported.

To test Hypotheses 6 and 7, which proposed that growth mindset moderates the indirect effect of using GAI on innovative behavior via effort expectancy and performance expectancy, the conditional indirect effects were further examined. As reported in Table 4, the index of moderated mediation (IMM) for the indirect effect of using GAI on innovative behavior via effort expectancy was not significant (IMM = 0.065, SE = 0.080, 95%CI = [−0.092, 0.223], including 0), indicating that the mediating effect through effort expectancy remained relatively stable across different levels of growth mindset. Thus, Hypothesis 6 was not supported. In contrast, the IMM for the indirect effect of using GAI on innovative behavior via performance expectancy was significant (IMM = −0.360, SE = 0.083, 95%CI = [−0.523, −0.198], excluding 0), suggesting that the indirect effect via performance expectancy varied according to students’ levels of growth mindset. Therefore, Hypothesis 7 was supported. Overall, the results suggested that the role of growth mindset might vary under different conditions, offering valuable directions for future research (see Table 5).

## 5. Discussion and Implications

Drawing on the UTAUT as the theoretical framework, this study constructs a moderated dual-mediation model by introducing effort expectancy and performance expectancy as mediating variables and growth mindset as a moderating variable. This study aims to examine how GAI use influences college students’ innovative behavior and to clarify the boundary conditions of GAI application in student learning contexts.

First, the direct effect of using GAI on innovative behavior is not significant, while the two indirect effects through effort expectancy and performance expectancy are both significantly positive. This finding indicates an indirect-only effect pattern. In this case, the influence of using GAI on innovative behavior appears to be transmitted primarily through indirect pathways rather than a direct pathway, which may explain the non-significant direct effect observed in the study. Therefore, the impact of using GAI on innovative behavior should be understood as a process mediated by specific psychological mechanisms rather than as a direct effect. Future research may further examine additional pathways through which using GAI influences innovative behavior to develop a more in-depth understanding of the effect pattern. Second, the use of GAI influences innovative behavior through two parallel pathways, namely effort expectancy and performance expectancy. Finally, this study identifies a significant negative moderating effect of growth mindset on the relationship between performance expectancy and innovative behavior. Specifically, the effect of performance expectancy on innovative behavior becomes weaker among students with a higher level of growth mindset. However, the moderating effect of growth mindset on the relationship between effort expectancy and innovative behavior is not significant. This may be because effort expectancy reflects students’ effort perceptions regarding GAI use, which relates to practical operational perceptions rather than the stable ability beliefs underlying growth mindset. As a result, growth mindset does not noticeably alter the relationship between effort expectancy and innovative behavior.

### 5.1. Theoretical Implications

First, this study may complement the literature on GAI in higher education by shifting the focus on adoption and usage intention. Prior studies have focused on students’ perceptions, perceived benefits, concerns, and acceptance of GAI, showing that learners often recognize its potential for personalized learning support, brainstorming, writing assistance, and productivity, while simultaneously worrying about accuracy, privacy, ethics, and academic integrity (Chan & Hu, 2023; X. Zhao et al., 2025). In that sense, the existing literature has mainly discussed whether students may be willing to use GAI and why they accept it (Strzelecki, 2024; Strzelecki et al., 2024). Instead, the study shifts attention to innovative behavior as a distal educational outcome. This perspective suggests that the value of GAI could extend beyond acceptance or usage intentions, and draws attention to understanding how students cognitively process their use experience and whether such experience may translate into innovation-related behavior.

Second, this study enriches the literature linking GAI use to creativity and productivity. Experimental and field studies in work and task settings have often reported that GAI can improve productivity or individual creativity, although such benefits are not necessarily uniform across different users, task types and practical application contexts (Noy & Zhang, 2023; Doshi & Hauser, 2024; Jia et al., 2024; S. Sun et al., 2025). Different from prior work that focuses on direct links between GAI and creative outcomes, this study identifies an indirect pathway connecting GAI use to students’ innovative behavior. This perspective helps clarify the cognitive processes through which GenAI use relates to innovative behavior in educational settings, and also complements existing findings by revealing the intermediate mechanisms behind such relationships.

Third, this study contributes to research on post-adoption technology use within the UTAUT framework. UTAUT was originally developed to explain technology adoption, in which performance expectancy and effort expectancy function as important antecedents of technology use (Venkatesh et al., 2003). Consequently, most UTAUT-based studies have focused on users’ adoption intentions and usage behaviors, including recent educational research examining college students’ acceptance and use of GAI (Yakubu et al., 2025; Tang et al., 2025). Although some scholars have begun to examine post-adoption contexts (Venkatesh et al., 2011; Singh, 2020), studies addressing post-adoption attitudes and behavioral outcomes remain considerably less developed than those examining technology adoption (Gupta et al., 2020). Building on this perspective, this study examines the role of expectancy beliefs in the post-adoption stage of GAI use. Specifically, it conceptualizes effort expectancy and performance expectancy as cognitive evaluations that may continue to be refined through ongoing interactions with GAI and the experiences accumulated through such continued use (Bhattacherjee, 2001). By examining how students’ actual engagement with GAI relates to these expectancy beliefs and subsequently to innovative behavior, the study provides a post-adoption perspective for understanding the cognitive processes associated with GAI use. Accordingly, UTAUT may be applied not only to explore factors influencing students’ adoption of GAI, but also to examine how usage experience alters students’ cognitive judgments and subsequent behavioral tendencies.

Fourth, the findings on moderating effects contribute additional insights to existing mindset research within GAI-assisted learning settings. Classic studies on growth mindset have illustrated that the belief in malleable abilities is closely linked to individuals’ self-regulation and goal engagement (Burnette et al., 2013; Dweck, 2006). Most relevant discussions focus on how growth mindset generally shapes people’s behavioral choices across various scenarios. By examining its moderating role in the relationship between cognitive perceptions and innovative behavior, this study contextualizes mindset within technology-based learning research. It further reveals that the influence of growth mindset does not operate uniformly when interacting with students’ perceptions of GAI. These observations do not challenge the core viewpoints of mindset theory. Instead, they help further understand how mindset traits function alongside GAI use, and elaborate on the joint influence of personal traits and technology cognition on students’ innovative behavior.

### 5.2. Practical Implications

First, college students should make more attempts at applying GAI in flexible learning tasks, rather than limiting its use to simple and routine tasks, like obtaining ready-made answers. Ideally, students can use GAI to brainstorm unconventional project ideas, design experimental schemes and revise drafts of academic work. These practices cultivate positive effort expectancy and performance expectancy, which lower barriers to creative thinking and reduce the cost of trial and error, thus enabling students to pursue innovative explorations.

Second, teachers should learn to collaborate with GAI, rather than being preoccupied with the fear of being replaced by GAI. For example, they can incorporate GAI into the teaching plan, allowing students to fully utilize GAI. Moreover, teachers can provide immediate feedback to students after their use of GAI, thereby strengthening the link between GAI use and students’ effort expectancy and performance expectancy.

Finally, colleges should introduce targeted GAI tools based on the disciplinary characteristics of different majors to enhance their practical applicability in learning contexts. For instance, STEM students can use GAI for data visualization and experimental design, while social science and humanities students can apply it to literature sorting and material summarization. This tailored integration helps translate routine GAI use into stronger positive expectancy beliefs, thereby encouraging students to engage in more innovative practices.

### 5.3. Limitations and Future Research

First, this study has certain limitations in measurement reliability and research design. The Cronbach’s alpha values of effort expectancy (α = 0.637) and performance expectancy (α = 0.665) are below the conventional threshold of 0.70. Although these values meet the acceptable standard for exploratory research (Hair et al., 2019), they still indicate relatively low stability of the measurement. This issue may be related to the cross-sectional design and the use of self-reported data, which may limit the objectivity of the results. To address these problems, future research will adopt multi-stage questionnaire distribution to enhance data quality and sample representativeness. Experimental designs will also be applied by assigning participants to two groups while holding relevant control variables constant. One group uses GAI during learning activities, and the other does not. Researchers then measure and compare students’ innovative behavior between the two groups, which helps reduce potential bias and further improves the rigor and credibility of the findings.

Second, some measurement scales in the study are integrated from multiple validated instruments, and satisfactory reliability has been achieved in the current sample. To further improve measurement rigor, future research will prioritize mature and well-established scales that are highly consistent with the target populations. If scale integration is still necessary to match specific research contexts, complete validation procedures, including confirmatory factor analysis and measurement invariance tests, will be carried out to ensure the effectiveness and reliability of the combined measures.

Third, the non-significant direct effect of GAI use on students’ innovative behavior needs further exploration. The direct effect of using GAI on students’ innovative behavior is not statistically significant, whereas the two mediating paths are positive. Although the present study contextualizes this finding through the identified indirect-only effect in the current sample, other cognitive factors, alternative mediating pathways and varied individual characteristics may alter the observed association across research settings. Future research will examine this issue by testing additional mediating and moderating variables and exploring other relevant conditions, thereby providing a more comprehensive understanding of the nuanced linkage between GAI use and students’ innovative behavior.

## 6. Conclusions

In conclusion, this study explored how the use of GAI relates to college students’ innovative behavior from the perspective of UTAUT. The findings indicate that GAI use is positively associated with college students’ effort expectancy and performance expectancy, which in turn support the development of their innovative behavior. Rather than exerting a direct influence, the relationship between GAI use and innovative behavior appears to operate primarily through these cognitive mechanisms. In addition, the association between performance expectancy and innovative behavior tends to be greater among students with lower levels of growth mindset.

Beyond these empirical findings, this study adds to existing research on GAI in higher education by focusing on post-adoption usage. By treating effort expectancy and performance expectancy as dynamic cognitive evaluations that develop through continued GAI engagement, the study presents a post-adoption perspective to explain the relationship between students’ GAI experiences and innovation-related outcomes. The findings further indicate that GAI’s educational value is associated with both students’ basic usage behavior and the long-term changes in their cognitive perceptions driven by continued use.

Given the growing presence of GAI in higher education, exploring how to translate AI-supported learning experiences into innovation-related outcomes remains a worthy direction for future research and educational practice.

## Figures and Tables

**Table 1:** Confirmatory factor analysis.

Model	*χ* ^2^	*df*	Δ*χ*^2^ (Δ*df*)	RMSEA	CFI	TLI	SRMR
Six-factor model (GAI; EE; PE; GM; IB; CMV)	246.359	140	101.412 (20) **	0.042	0.959	0.944	0.034
Five-factor model (GAI; EE; PE; GM; IB)	347.771	160	/	0.052	0.927	0.913	0.052
Four-factor model (GAI; EE + PE; GM; IB)	444.813	164	97.042 (4) **	0.063	0.891	0.873	0.057
Three-factor model (GAI; EE + PE + GM; IB)	787.729	167	439.958 (7) **	0.093	0.758	0.725	0.080
Two-factor model (GAI + EE + PE + GM; IB)	945.285	169	597.514 (9) **	0.103	0.698	0.660	0.090
One-factor model (GAI + EE + PE + GM + IB)	1237.874	170	890.103 (10) **	0.121	0.584	0.535	0.104

Note: ** *p* < 0.01. CMV: common method variance factor; GAI = Use of GAI; EE = Effort expectancy; PE = Performance expectancy; GM = Growth mindset; IB = Innovative behavior.

**Table 2:** Means, standard deviations, and correlations among study variables.

Variables	Mean	SD	1	2	3	4	5	6	7	8
1. Gender	1.50	0.50								
2. Age	22.17	1.94	−0.120 *							
3. Educational level	1.26	0.47	−0.077	0.601 **						
4. Major	7.19	3.14	−0.061	0.103 *	0.055					
5. Use of GAI	5.49	0.56	−0.055	0.030	0.012	−0.046				
6. Effort expectancy	5.93	0.52	−0.139 **	0.083	0.048	0.010	0.286 **			
7. Performance expectancy	6.00	0.50	−0.085	0.064	−0.017	0.001	0.391 **	0.367 **		
8. Innovative behavior	5.39	0.83	−0.160 **	0.078	−0.134 **	−0.003	0.208 **	0.313 **	0.322 **	
9. Growth mindset	5.31	0.85	−0.014	0.010	−0.046	−0.088	0.156 **	0.176 **	0.181 **	0.443 **

Note: * *p* < 0.05; ** *p* < 0.01.

**Table 3:** Direct and indirect effects.

Path	Effect	SE	95%CI
*Direct effect*			
Use of GAI → innovative behavior	−0.190	0.120	[−0.426, 0.045]
Use of GAI → effort expectancy	0.492	0.076	[0.342, 0.641]
Use of GAI → performance expectancy	0.413	0.069	[0.278, 0.548]
*Indirect effect*			
Use of GAI → effort expectancy → innovative behavior	0.157	0.053	[0.053, 0.261]
Use of GAI → performance expectancy → innovative behavior	0.288	0.078	[0.135, 0.440]

**Table 4:** Interaction and moderated mediation effects.

Path	Effect	SE	95%CI
*Interaction effect*			
Effort expectancy × growth mindset → innovative behavior	0.133	0.161	[−0.183, 0.449]
Performance expectancy × growth mindset → innovative behavior	−0.872	0.202	[−1.268, −0.477]
*Moderated mediation effect*			
Use of GAI → effort expectancy × growth mindset → innovative behavior	0.065	0.080	[−0.092, 0.223]
Use of GAI → performance expectancy × growth mindset → innovative behavior	−0.360	0.083	[−0.523, −0.198]

**Table 5:** Indirect effect of performance expectancy on innovative behavior at different levels of growth mindset.

Path	Growth Mindset	Indirect Effect	SE	95%CI
GAI → PE → IB	+1 SD	−0.02	0.091	[−0.199, 0.159]
	−1 SD	0.595	0.117	[0.365, 0.825]
	Difference	−0.615	0.141	[−0.892, −0.337]

Note: GAI = Use of GAI; PE = Performance expectancy; IB = Innovative behavior.

## Data Availability

The datasets of the current study are available upon request from the corresponding author due to data management policies of the affiliated institution.

## Appendix A

**Items for measuring our variables**

***Use of GAI*** (source: Y. Li et al. (2024) and Abbas et al. (2024))

1. I use generative AI to answer questions posed by the teacher in class.

2. I use generative AI to look up information related to the course content.

3. I use generative AI to assess coursework and provide feedback.

4. I use generative AI for my course assignments.

5. I use generative AI for my academic activities.

6. I use generative AI to prepare for my tests or quizzes.

7. I use generative AI for my course projects.

8. I use generative AI to learn course-related concepts.

9. I rely on generative AI for my studies.

10. I am addicted to generative AI when it comes to studies.

11. Generative AI is part of my campus life.

***Innovative behavior*** (source: Scott & Bruce, 1994)

1. Searches out new technologies, processes, techniques, and/or product ideas.

2. Generates creative ideas.

3. Promotes and champions ideas to others.

4. Investigates and secures the necessary resources and support needed to implement new ideas.

5. Develops adequate plans and schedules for the implementation of new ideas.

6. Is innovative.

***Growth mindset*** (source: Sigmundsson and Haga (2024) and Xiao et al. (2026))

1. Intelligence is something you can increase substantially.

2. You can always change your basic level of intelligence.

3. No matter how smart you are, you can always get smarter.

4. Learning new things does not change your intelligence.

5. You have a certain amount of intelligence, and you can’t really change it.

6. Trying new things is a way to grow your intelligence.

7. Challenges help you develop your intelligence.

8. Effort is what makes you smarter.

***Effort expectancy*** (source: Cao et al., 2021)

1. Learning how to use generative AI is easy for me.

2. My interaction with generative AI is clear and understandable.

3. I find generative AI easy to use.

4. It is easy for me to become skillful at using generative AI.

***Performance expectancy*** (source: Cao et al., 2021)

1. I find generative AI useful in my decision-making.

2. Using generative AI increases my chances of making important decisions.

3. Using generative AI helps me make decisions more quickly.

4. Using generative AI increases my productivity.

5. Using generative AI increases my productivity in decision-making.
